# Exploring the Biological Potency of Carotenoids Against Alzheimer’s Disease: An Integrated Approach of Molecular Docking and Molecular Dynamics

**DOI:** 10.3390/cimb48040407

**Published:** 2026-04-16

**Authors:** Meriem Khedraoui, El Mehdi Karim, Imane Yamari, Abdelkbir Errougui, Doni Dermawan, Nasser Alotaiq, Samir Chtita

**Affiliations:** 1 Laboratory of Analytical and Molecular Chemistry, Faculty of Sciences Ben M’Sick, Hassan II University of Casablanca, Casablanca 20670, Morocco; meriemkhedraoui5@gmail.com (M.K.); 2013karim.mehdi@gmail.com (E.M.K.); yamariimane86@gmail.com (I.Y.); a_errougui@yahoo.fr (A.E.); samirchtita@gmail.com (S.C.); 2 Department of Applied Biotechnology, Faculty of Chemistry, Warsaw University of Technology, 00-661 Warsaw, Poland; doni.dermawan.stud@pw.edu.pl; 3 Health Sciences Research Center (HSRC), Imam Mohammad Ibn Saud Islamic University (IMSIU), Riyadh 13317, Saudi Arabia

**Keywords:** Alzheimer’s disease, carotenoids, Hopkinsiaxanthin, molecular docking, molecular dynamics, MM/PBSA, pharmacophore, per-residue decomposition

## Abstract

Alzheimer’s disease (AD) is a multifactorial neurodegenerative disorder characterized by cholinergic dysfunction, amyloid-β aggregation, mitochondrial stress, and aberrant kinase activity. Carotenoids, naturally occurring pigments with antioxidant and neuroprotective properties, have emerged as promising candidates for AD intervention. In this study, we performed a systematic stepwise computational screening of a large carotenoid library (*n* = 1191) to identify multitarget candidates against AD–related proteins. The workflow consisted of predefined ADMET filtering (oral absorption > 90%, Caco-2 > 0.9, logBB > −1, and absence of major CYP inhibition and toxicity alerts), reducing the dataset to 61 compounds, followed by multi-target molecular docking against AChE, BChE, BACE-1, MAO-B, and GSK3-β. Compounds were ranked using an aggregated mean docking score across all five targets, and the top-performing candidate was subjected to detailed mechanistic analyses. Hopkinsiaxanthin emerged as the highest-ranked multitarget carotenoid and was further evaluated using frontier molecular orbital (FMO) analysis, pharmacophore modeling, 100 ns molecular dynamics (MD) simulations, MM/PBSA binding free energy calculations, and per-residue decomposition. Docking predicted favorable estimated binding affinities toward all targets. MD simulations confirmed stable receptor–ligand complexes with low RMSD values (0.278–0.285 nm). MM/PBSA analysis indicated favorable binding free energies, particularly for GSK3-β (−22.73 kcal/mol) and AChE (−21.50 kcal/mol). Per-residue decomposition identified key hotspot residues driving stabilization. Overall, this structured computational framework identifies Hopkinsiaxanthin as a promising multitarget scaffold and supports its prioritization for experimental validation in AD models.

## 1. Introduction

Alzheimer’s disease (AD) is a prototypical neurodegenerative disorder affecting the central nervous system (CNS). AD leads to a deterioration of cognitive ability, behavioral changes, and functional abilities linked to aging. AD is the most common form of dementia in older adults and is considered the leading cause of death [1]. AD constitutes a major public health problem, raising many concerns. This has been considered a research priority [2]. There are millions of people affected by AD globally. The number of people with dementia is estimated at more than 50 million and will reach 150 million by 2050 [3,4]. However, the etiology of AD is not fully understood and remains unclear and uncertain to this day [5]. AD is a complex neurodegenerative disorder characterized by the progressive loss of neurons and synapses, which disrupts neural connectivity and leads to cognitive decline [6]. Although its etiology is multifactorial [7], several mechanisms have been proposed, including the extracellular accumulation of β-amyloid peptide (Aβ), resulting from abnormal cleavage of the amyloid precursor protein (APP) by β-site amyloid precursor protein cleaving enzyme 1 (BACE-1), leading to the formation of senile plaques [8], as well as the hyperphosphorylation of tau protein, is responsible for neurofibrillary tangles. In parallel, dysfunction of the cholinergic system, particularly a decrease in acetylcholine levels in the hippocampus, has been widely reported [9], partly due to increased acetylcholinesterase (AChE) activity [10]. Neuroinflammation involving activated microglia, neutrophils, and macrophages has emerged as a key factor in disease progression [11]. Currently available treatments, such as cholinesterase inhibitors (rivastigmine, donepezil, galantamine), target symptoms but do not halt disease progression [12,13,14,15]. This highlights the need to explore new therapeutic strategies targeting amyloid, tau-related, inflammatory, and oxidative processes [16].

Oxidative stress, an imbalance between the production of reactive oxygen species (ROS) and antioxidant defense mechanisms, can damage biomolecules and promote chronic inflammation. It plays a central role in both brain aging and the pathological process of AD. Elevated levels of oxidative stress have been observed not only in the brain but also in the peripheral tissues of patients with AD. This imbalance is characterized by excessive ROS production, dysfunction of antioxidant systems, and increased oxidative damage across various brain regions, including in individuals with mild cognitive impairment (MCI) [17,18,19,20]. Inflammation is a normal physiological response triggered by the body to harmful stimuli to restore tissue integrity and maintain internal balance. Although initially beneficial by supporting cellular repair and eliminating toxic substances, it is now well established that chronic inflammation plays a key role in the development of neurodegenerative diseases, including AD. As the disease progresses, this ongoing inflammatory response becomes harmful and directly contributes to neurodegeneration [21,22,23,24,25].

The primary sources of compounds essential for therapeutic development are mainly natural products with the potential to serve as adjuvant therapies to relieve the symptoms of AD, including medicinal plants [26]. Recently, scientific research has focused on carotenoid compounds due to their biological effects against AD and their natural occurrence. Carotenoids are generally tetraterpenoid pigments synthesized by most plants, some bacteria, and fungi [27]. They possess a series of conjugated double bonds that form their chromophore, responsible for the bright red, orange, and yellow colors observed in various organisms [28]. There are two types of carotenoids: carotenes and xanthophylls. Carotenoids are hydrocarbon compounds that do not contain any substituents or oxygen, while xanthophylls are molecules that contain oxygen [29]. Many biological functions have been attributed to carotenoids, including acting as provitamin A precursors, activating nuclear hormone receptors and the immune system, serving as molecular signals, as well as acting as antioxidants and anti-inflammatories [30]. Regular consumption of foods containing carotenoids is associated with a reduced risk of heart disease, cancer, macular degeneration, and other eye conditions [31]. Recent research has highlighted the importance of carotenoids in treating neurodegenerative disorders. Indeed, carotenoids are characterized by potent antioxidant and anti-inflammatory properties, which are particularly significant in AD, where oxidation and neuroinflammation play a substantial role in disease progression. Numerous in vitro and in vivo studies have shown that these molecules can reduce β-amyloid peptide accumulation and tau protein hyperphosphorylation, helping slow neurodegeneration [32,33]. For example, astaxanthin and fucoxanthin have demonstrated their ability to cross the blood–brain barrier, resulting in a direct neuroprotective effect [34]. These advances position carotenoids as potential adjuvant strategies, although further clinical trials are still required to confirm their efficacy in humans.

Given the multifactorial nature of AD and the emerging therapeutic potential of carotenoids, this study was designed to systematically evaluate a comprehensive dataset of naturally occurring carotenoids for their inhibitory potential against five key AD-associated targets. A stepwise computational screening workflow was employed to achieve this, encompassing absorption, distribution, metabolism, excretion, and toxicity (ADMET)-based pharmacokinetic and drug-likeness profiling, molecular docking, density functional theory-based frontier orbital analysis, pharmacophore modeling, and molecular dynamics simulations. Furthermore, binding free energies were quantified using the Molecular Mechanics/Poisson–Boltzmann Surface Area (MM/PBSA) approach, complemented by per-residue free energy decomposition to identify critical amino acid residues contributing to binding stability. Collectively, this study aims to identify carotenoid scaffolds with favorable pharmacological profiles and vigorous multi-target inhibitory activity, thereby supporting their rational development as potential therapeutic candidates for AD.

## 2. Materials and Methods

### 2.1. Carotenoids Dataset

A comprehensive dataset of carotenoid compounds was retrieved from the publicly available Carotenoid Database (http://carotenoiddb.jp, accessed on 14 March 2025), which serves as a curated repository for carotenoid structures and related information. Each compound corresponds to a unique Carotenoid Database ID; although these IDs were not explicitly listed in Supplementary Data S1, they can be accessed directly and verified through the official database entry list (http://carotenoiddb.jp/Entries/list1.html, accessed on 14 March 2025). This ensures unambiguous structural traceability, particularly in cases where identical compound names correspond to different structural isomers with distinct SMILES representations. Stereochemical information was systematically evaluated for all carotenoids prior to 3D model construction. When stereocenters (e.g., hydroxyl-bearing chiral carbons in xanthophyll-type carotenoids such as Hopkinsiaxanthin or Cochloxanthin) were not explicitly annotated in the 2D database depiction, the configuration was assigned based on the stereochemical descriptors available in the original database record or associated literature, and subsequently verified during 3D structure generation. Thus, all computational calculations were conducted using fully specified 3D structures with explicitly defined chiral centers and complete oxygen atom representations, even if such details are not visually emphasized in the simplified 2D drawings shown in Table 1. These compounds were selected with the objective of evaluating their inhibitory potential against five key protein targets implicated in the pathogenesis of AD: AChE, butyrylcholinesterase (BChE), BACE-1, monoamine oxidase (MAO), and glycogen synthase kinase-3 beta (GSK3-β). Initially, the two-dimensional (2D) chemical structures of the carotenoids were either directly obtained from the database or redrawn for accuracy using ChemDraw version 22 (PerkinElmer, Waltham, MA, USA) [35]. These 2D structures were then converted into their corresponding three-dimensional (3D) conformations using the Chem3D Ultra version 22 (PerkinElmer, Waltham, MA, USA) [35], where energy minimization was performed to ensure geometrical optimization and stability of the molecular structures. The energy minimization step employed the MM2 force field to refine bond lengths, bond angles, torsional strain, and non-bonded interactions, thereby generating energetically favorable conformations suitable for downstream computational studies. The optimized 3D structures were saved in Protein Data Bank (PDB) format, which is compatible with molecular docking and molecular dynamics simulation platforms.

### 2.2. In Silico ADMET Predictions

ADMET profiling represents a fundamental aspect of pharmacokinetics, providing critical insights into how a compound behaves within a biological system. These parameters determine candidate molecules’ bioavailability, safety, and therapeutic efficacy. Specifically, ADMET analyses help assess systemic distribution, metabolic stability, elimination pathways, and potential toxicological risks, thereby guiding the identification of compounds with favorable drug-like characteristics and minimizing late-stage drug development failures [36]. This study systematically predicted ADMET properties and Lipinski’s rule of five descriptors to evaluate the pharmacological potential of carotenoid compounds. The SMILES notations of the selected molecules were first generated and used as input files for the computational analyses. Predictions of ADMET properties, including human intestinal absorption (HIA), BBB permeability, CNS permeability, cytochrome P450 (CYP) enzyme interactions, and toxicity endpoints, were carried out using the pkCSM pharmacokinetics server (https://biosig.lab.uq.edu.au/pkcsm/, accessed on 12 April 2025) [37]. In parallel, drug-likeness and physicochemical properties, such as molecular weight, lipophilicity (LogP), hydrogen bond donors and acceptors, and topological polar surface area (TPSA), were evaluated using the SwissADME web server (http://www.swissadme.ch/, accessed on 12 April 2025) [38]. This two-step computational approach allowed for the filtering of compounds with poor pharmacokinetic or physicochemical profiles, ensuring that only molecules with favorable oral bioavailability, metabolic stability, and low predicted toxicity were retained for further molecular docking and molecular dynamics simulations.

### 2.3. Molecular Docking Simulations and Binding Affinity Analysis

Molecular docking studies were conducted to investigate the binding mechanisms of carotenoid compounds with AD-associated target proteins and to predict the most favorable ligand orientations and conformations within the binding pockets [39]. This approach allowed the identification of potential inhibitory activity and facilitated the characterization of key molecular interactions. The high-resolution 3D crystal structures of the selected targets: AChE (PDB ID: 4EY7 [40]), BChE (PDB ID: 7AIY [41]), BACE-1 (PDB ID: 3UQU [42]), MAO (PDB ID: 2Z5X [43]), and GSK3-β (PDB ID: 1J1B [44]), were retrieved from the RCSB Protein Data Bank. Protein structures were pre-processed by removing crystallographic water molecules, ions, and non-essential ligands, optimizing protonation states, and adding polar hydrogens to ensure proper electrostatic interactions during docking. Correct protonation states of ionizable amino acid residues were assigned, assuming physiological pH (7.4), using structure-based pKa estimation and hydrogen-bond network inspection, ensuring chemically consistent charge states for catalytic residues and binding-site side chains. Ligands were protonated according to their dominant microspecies at pH 7.4 prior to docking and MD simulations. For MAO-B, the flavin adenine dinucleotide (FAD) cofactor, which is essential for catalytic activity and structural integrity of the active site, was retained during receptor preparation and included in all docking calculations to preserve the native binding environment. Only non-essential crystallographic components were removed.

Ligand preparation involved conversion of structures into AutoDock-compatible formats, ensuring correct protonation states and energy minimization. The docking procedure was performed using AutoDock Vina version 1.2.0 (The Scripps Research Institute, La Jolla, CA, USA) [45,46], selected for its robust scoring function and reliable prediction of protein–ligand binding modes. A grid box was centered on the co-crystallized ligand binding site to ensure physiologically relevant docking. For AutoDock Vina, the search space was defined by grid box dimensions of 40 × 40 × 40 Å (center_x, center_y, center_z specified according to the native ligand coordinates). No grid spacing parameter was defined, as Vina internally uses its own grid representation. Docking calculations were carried out using the Vina iterated local search global optimizer, with an exhaustiveness value of 8, num_modes set to 10, and a default energy_range of 3 kcal/mol to ensure adequate conformational sampling. To validate the docking protocol, re-docking of the co-crystallized ligands was performed for representative targets (AChE, BACE-1, and GSK3-β) using identical parameters. The re-docked poses reproduced the experimental binding conformations with root-mean-square deviation (RMSD) values below 2.0 Å, confirming the reliability and reproducibility of the docking setup. For example, AChE (PDB ID: 4EY7), originally co-crystallized with donepezil, yielded a re-docked pose consistent with the native binding orientation. Multiple binding poses were generated for each ligand–protein pair, and the pose with the lowest estimated binding affinity (kcal/mol) was selected as the optimal conformation. Post-docking interaction analysis was carried out using Discovery Studio Visualizer 2024 (Dassault Systèmes BIOVIA, San Diego, CA, USA) [47] to characterize hydrogen bonds, hydrophobic interactions, van der Waals forces, and other non-covalent interactions contributing to binding affinity. Reference ligands were selected based on target specificity and literature validation. Where available, the co-crystallized inhibitor for each PDB structure was used as the primary comparator. Additional reference drugs (e.g., donepezil and tacrine for cholinesterases, rivastigmine for dual cholinesterase inhibition, and resveratrol for BACE-1-related studies) were included only where mechanistically relevant and supported by experimental evidence.

### 2.4. HOMO–LUMO Analysis of the Best Carotenoid

The top-performing carotenoid’s frontier molecular orbital (FMO) properties were investigated using density functional theory (DFT) calculations. All molecular geometries were fully optimized at the PBE0/6-31G(d) level of theory. The PBE0 hybrid functional, originally introduced by Adamo and Barone, incorporates 25% exact Hartree–Fock exchange into the Perdew–Burke–Ernzerhof (PBE) exchange–correlation functional and has been widely validated for reliable prediction of molecular geometries, thermochemistry, and frontier orbital energies [39]. Its balanced treatment of exchange and correlation makes it suitable for evaluating electronic descriptors of conjugated systems such as carotenoids. Convergence criteria were set to tight self-consistent field (SCF) thresholds, with an energy tolerance of 10^−6^ Ha and a maximum gradient of 10^−3^ Ha/Bohr, ensuring accurate and stable optimization [48]. Following geometry optimization, single-point energy calculations were performed to determine orbital eigenvalues. The highest occupied molecular orbital (HOMO) and lowest unoccupied molecular orbital (LUMO) were automatically identified, and the corresponding energy gap (ΔE = E_LUMO − E_HOMO) was computed. This energy gap is a key descriptor of molecular stability, electronic excitation potential, and overall chemical reactivity [49]. To assess electron density distribution, HOMO and LUMO iso-surfaces were generated using standard visualization tools, with an iso-value of 0.02 a.u. [50,51]. This enabled direct evaluation of orbital delocalization and highlighted the structural regions most involved in electronic transitions. By providing consistent and reproducible electronic descriptors, this computational protocol facilitated comparison of the best carotenoid’s reactivity profile concerning AD therapeutic targets. The results support a deeper understanding of how electronic features may contribute to ligand–target binding affinity and stability.

### 2.5. 3D Pharmacophore Modeling

Three-dimensional (3D) pharmacophore modeling was carried out to identify and characterize the essential molecular features responsible for the interactions between the best carotenoid compound and AD-associated target proteins (AChE, BChE, BACE-1, MAO, and GSK3-β). This approach provides a structural framework for understanding how specific chemical functionalities influence receptor recognition and binding affinity. Pharmacophore models were generated using LigandScout version 4.5 (Inte:Ligand GmbH, Vienna, Austria) [52], which automatically detects and maps critical interaction features, including hydrogen bond donors (HBDs), hydrogen bond acceptors (HBAs), hydrophobic moieties, aromatic rings, and electrostatic interaction centers. These features represent the key determinants of ligand–protein binding specificity and stability. The analysis further provided insights into how specific carotenoid scaffolds could be optimized to improve multi-target inhibitory activity, thereby supporting the rational design of carotenoid-based therapeutic candidates for AD.

### 2.6. Molecular Dynamics (MD) Simulation for Structural Stability and Interaction Analysis

The MD simulations were employed to validate the reliability of the molecular docking results and to investigate the conformational stability, dynamic behavior, and interaction mechanisms of the selected carotenoid–target protein complexes. All simulations were carried out using GROMACS version 2025.2 [53,54]. A consistent CHARMM-based parameterization scheme was employed, using the CHARMM36m force field for proteins and CGenFF (CHARMM General Force Field) parameters for ligands, ensuring full compatibility within the same force-field ecosystem. Ligand parameters were generated using the CGenFF program and converted into GROMACS-compatible topologies. The protein–ligand complexes were solvated in a dodecahedral periodic box filled with the TIP3P water model, maintaining a minimum distance of 1.0 nm between the solute and the box boundaries. To neutralize the system and emulate physiological conditions, Na^+^ and Cl^−^ counterions were introduced to achieve a final ionic concentration of 0.15 M. Energy minimization was conducted using the steepest descent algorithm until the maximum force was reduced below 1000 kJ/mol/nm, ensuring removal of steric clashes [55]. The equilibrated system was subjected to two successive steps: (i) constant volume (NVT) equilibration for 100 ps using a V-rescale thermostat to maintain the temperature at 300 K, and (ii) constant pressure (NPT) equilibration for 100 ps using the Parrinello–Rahman barostat to stabilize pressure at 1 bar [56,57,58]. During equilibration, position restraints were applied to the protein heavy atoms to prevent structural distortions. Subsequently, unrestrained production MD simulations were performed for 100 ns with an integration time step of 2 fs. Long-range electrostatic interactions were calculated using the Particle Mesh Ewald (PME) method, and van der Waals interactions were truncated at 1.0 nm. Trajectories were recorded every 10 ps for post-simulation analysis. Comprehensive trajectory analyses were carried out to evaluate the structural stability and binding dynamics of the complexes [59,60,61]. The following parameters were calculated: RMSD (reported in nm) for overall system stability, RMSF (reported in nm) for residue-level flexibility, RoG (reported in nm) to assess protein compactness, SASA (reported in nm^2^) for hydration dynamics, and hydrogen bond profiles to monitor intermolecular interactions. Interaction profiles were analyzed using PyMOL version 3.1.3 (Schrödinger LLC, New York, NY, USA) [62], BIOVIA Discovery Studio 2024 (Dassault Systèmes, San Diego, USA) [47], and UCSF ChimeraX (University of California, San Francisco, CA, USA) [63], enabling both quantitative measurements and qualitative visualization of the ligand–protein dynamics.

### 2.7. Molecular Mechanics/Poisson–Boltzmann Surface Area (MM/PBSA) Calculations

The binding free energies of the top-ranked carotenoid in complex with AD–related protein targets (AChE, BChE, BACE-1, MAO, and GSK3-β) were estimated using the MM/PBSA approach. This method integrates molecular mechanics energies with continuum solvation models to comprehensively evaluate protein–ligand binding affinities [64]. A total of 500 snapshots were uniformly extracted from the equilibrated portion of each trajectory, specifically from the final 50 ns of the 100 ns production run, at regular 100 ps intervals (i.e., one frame every 100 ps), ensuring statistically robust and reproducible sampling. For each snapshot, three principal energetic components were evaluated: (i) Molecular mechanics energy in the gas phase, encompassing van der Waals and electrostatic interactions; (ii) Solvation free energy, consisting of a polar contribution calculated via the Poisson–Boltzmann (PB) continuum model and a nonpolar contribution estimated from the solvent-accessible surface area (SASA); (iii) Entropic contributions (−TΔS) were not calculated in this study (no normal mode or quasi-harmonic analysis was performed), and thus were explicitly neglected in the binding free energy estimation. Accordingly, the reported ΔG_binding values correspond to enthalpic terms only (ΔE_MM + ΔG_solv) and should be interpreted as relative binding energy estimates suitable for comparative ranking rather than absolute thermodynamic quantities. The Single-Trajectory Protocol (STP) was employed, whereby free energies of the complex, receptor, and ligand were derived from the same MD trajectory. This reduces computational cost and enhances consistency by assuming minimal structural rearrangements upon ligand binding [65,66]. The binding free energy (ΔG_binding) was computed using the thermodynamic cycle:

ΔG_binding = ΔG_complex − ΔG_ligand − ΔG_receptor(1)

where ΔG_complex is the total free energy of the protein–ligand complex, ΔG_ligand is the free energy of the unbound ligand in solution, and ΔG_receptor is the free energy of the isolated receptor. All MM/PBSA analyses were carried out using gmx_MMPBSA version 1.6.4, integrated with the GROMACS simulation framework [67]. The polar solvation energy was computed using the PB model with default dielectric constants (solute dielectric ε = 2, solvent dielectric ε = 80), and the mbondi atomic radii set was applied as implemented in gmx_MMPBSA. Apart from the explicitly stated parameters, default settings of the software were used. In addition, per-residue free energy decomposition was performed to identify key amino acid residues contributing significantly to ligand binding. Residue-wise decomposition (idecomp = 1) was applied, with verbose energy reporting enabled (dec_verbose = 1). Only residues within 6 Å of the ligand were included in the decomposition analysis (print_res = “within 6”), and results were exported in CSV format (csv_format = 1) for quantitative comparison. This residue-level analysis highlighted hydrogen bonding, hydrophobic contacts, and electrostatic hotspots within the binding pockets. It provided detailed insights into the molecular determinants of potent and selective interactions between carotenoid and its respective AD–related targets. The ΔG_binding values and per-residue energy profiles enabled a quantitative comparison across different carotenoids, thereby supporting the rational identification of structural features that enhance binding affinity and stability.

## 3. Results

### 3.1. Carotenoid Dataset Characterization

A total of 1191 carotenoid compounds were retrieved from the Carotenoid Database and structurally curated for computational analyses. These compounds encompass a diverse range of hydrocarbon carotenoids and oxygenated xanthophylls, reflecting the structural heterogeneity naturally occurring across plants, algae, fungi, and bacteria. To illustrate this diversity, a subset of ten representative carotenoids is presented in Table 1, along with their corresponding simplified molecular-input line-entry system (SMILES) notations and two-dimensional (2D) structural depictions. This representative set includes hydrocarbon derivatives such as Hopkinsiaxanthin, oxygenated analogs like Neurosporaxanthin and Tangeraxanthin, as well as specialized apocarotenoids such as 3-Hydroxy-β-apo-8′-carotenoic acid and Apoastacenal.

The inclusion of these representative molecules highlights the chemical space covered in this study, which ranges from highly conjugated linear carotenoids to cyclic structures with functional hydroxyl, carbonyl, and carboxyl substituents. These variations in molecular architecture directly influence carotenoid solubility, electronic properties, and biological activities, thereby providing a robust foundation for further pharmacokinetic and interaction analyses. The selected representatives also demonstrate the structural motifs hypothesized to be most relevant for interaction with AD–related protein targets. For example, hydroxylated carotenoids such as Aesculaxanthin and 3-Hydroxysintaxanthin exhibit enhanced polarity, potentially favoring hydrogen bonding interactions within active sites. Meanwhile, conjugated aldehydes like 12′-Apoastaxanthinal may present reactive centers facilitating π–π stacking with aromatic residues of target proteins. The complete dataset, provided in Supplementary Data S1, ensures that the computational screening was not limited to a narrow structural subset but spanned the full breadth of carotenoid diversity.

### 3.2. In Silico ADMET Profiling of Carotenoid Compounds

The ADMET profiling of the carotenoid library was performed to assess their drug-likeness and suitability as potential therapeutic candidates against AD. A total of 1191 carotenoid compounds were initially screened using predefined pharmacokinetic and toxicity thresholds. Compounds failing any of these essential safety or CNS-relevance criteria were excluded to reduce the risk of late-stage drug development failure. As summarized in Supplementary Data S2, of the 1191 screened molecules, 1130 compounds were eliminated during ADMET filtering, primarily due to excessive lipophilicity (LogP > 10), poor predicted BBB penetration (logBB < −1.0), subthreshold logPS values (<−2.0), or predicted toxicity/CYP inhibition flags. After sequential application of all criteria, 61 carotenoid compounds satisfied the defined pharmacokinetic and safety thresholds and were prioritized for further docking simulations. The majority of carotenoids did not fully comply with Lipinski’s Rule of Five (RO5), particularly due to their high lipophilicity (LogP > 5) and relatively large molecular sizes. Nonetheless, many compounds satisfied the molecular weight criterion (<500 Da), suggesting a subset of molecules with more favorable pharmacokinetic properties. Since lipophilicity strongly influences solubility and membrane permeability, these findings highlight the potential need for formulation strategies such as nanocomminution or lipid-based carriers to enhance the bioavailability of carotenoids with high LogP values [68]. The five representative carotenoids (Hopkinsiaxanthin, Neurosporaxanthin, Tangeraxanthin, Aesculaxanthin, and 3-Hydroxy-β-apo-8′-carotenoic acid) were further evaluated in detail to illustrate these ADMET properties (Table 2). Their molecular weights ranged between 448.6 and 498.7 Da, which falls within the acceptable limit set by Lipinski’s RO5, suggesting that these molecules are not excessively large and could theoretically be orally bioavailable. However, their lipophilicity values were consistently high (LogP = 6.709–10.110), exceeding the recommended threshold of 5. Elevated LogP values indicate poor aqueous solubility but strong affinity for lipid membranes, which may facilitate permeability while compromising solubility. Such characteristics imply that while these carotenoids can readily partition into biological membranes, specialized formulation approaches, such as nanoemulsions or lipid-based carriers, would be necessary to improve their dissolution and oral bioavailability.

Hydrogen bonding properties also play a critical role in solubility and target interactions. All compounds exhibited low numbers of hydrogen bond acceptors (HBA: 1–3) and donors (HBD: 1–2), which fall within the acceptable ranges of drug-likeness criteria. This balance suggests that the compounds are unlikely to face polarity-related absorption barriers. The Caco-2 permeability values further support this, as all compounds showed values above 1.0, surpassing the 0.9 threshold for high permeability [69]. Likewise, intestinal absorption values were consistently above 90%, indicating that these carotenoids are likely to be efficiently absorbed in the gastrointestinal tract. The distribution profiles of the compounds were particularly relevant in the context of AD, which requires CNS activity. The logBB values of the representative carotenoids ranged from −0.069 to −0.382, suggesting modest penetration across the blood–brain barrier (BBB). While none reached the optimal logBB > 0.3 threshold for strong BBB permeability, their ability to maintain measurable CNS distribution (logPS = −1.277 to −1.610) indicates that these carotenoids may still reach the brain at pharmacologically meaningful levels. Those with a logBB value lower than −1 are poorly distributed and will therefore be excluded from the study [70]. Despite their modest BBB penetration, their consistently high intestinal absorption and systemic bioavailability may compensate for limited CNS permeability, especially when paired with delivery strategies that enhance brain targeting.

Metabolic stability was assessed through cytochrome P450 (CYP) profiling. All five carotenoids were identified as substrates of CYP3A4, but not CYP2D6, and none exhibited inhibitory activity toward CYP2D6 or CYP3A4. This profile is favorable because it indicates efficient metabolism without posing risks of drug–drug interactions through CYP inhibition. Their total clearance values, ranging from 1.163 to 1.411, reflect moderate metabolic elimination, which is desirable for sustaining therapeutic levels without excessive drug accumulation. Toxicity predictions further supported the drug candidacy of these compounds. All exhibited high LD_50_ values (1.762–2.478 g/kg), placing them in a relatively safe toxicity class. None showed mutagenicity in the Ames test, hepatotoxicity, or skin sensitization risks. These findings are particularly significant since toxicity remains one of the primary causes of drug development attrition [71]. Moreover, the absence of such toxicity signals aligns with the known dietary safety of carotenoids, which have long been consumed as part of human nutrition. After applying ADMET-based filtering, 61 carotenoid molecules were prioritized for further analysis, demonstrating the most favorable pharmacokinetic and safety profiles. Given their acceptable oral absorption, moderate-to-good CNS permeability, safe toxicity characteristics, and lack of CYP-mediated metabolic inhibition, these compounds are promising for neuroprotective drug development.

### 3.3. Molecular Docking Results, Binding Pose, and Binding Affinity Analysis

**Figure 1:**
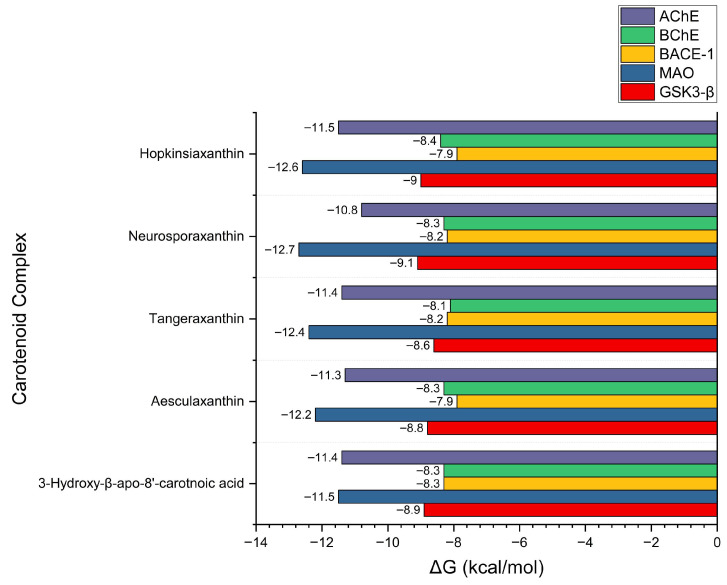
Binding affinity (ΔG, kcal/mol) of top-performing carotenoids against AD–related receptor targets (AChE, BChE, BACE-1, MAO, and GSK3-β).

Molecular docking was conducted to investigate the interaction potential of carotenoid compounds against five AD–related protein targets. Following ADMET-based screening, 61 carotenoids were selected and subjected to docking simulations, with binding energies compared to those of clinically relevant reference drugs. As shown in Supplementary Data S3 and visualized in Figure 1 for the representative, the carotenoids demonstrated binding affinities ranging from −5.5 to −12.7 kcal/mol across the tested targets, which in several cases exceeded the docking scores of the reference drugs. For example, donepezil, tacrine, resveratrol, and rivastigmine exhibited binding energies between −6.2 and −11.4 kcal/mol. In contrast, Hopkinsiaxanthin displayed consistently stronger binding scores, spanning −7.9 to −12.6 kcal/mol, indicating its potential as a multi-target modulator. Hopkinsiaxanthin emerged as the most promising candidate, exhibiting superior or comparable docking scores against all five proteins. Notably, its affinity for AChE (−11.5 kcal/mol) and MAO (−12.6 kcal/mol) surpassed that of the respective reference inhibitors, suggesting strong inhibitory potential. Neurosporaxanthin and Tangeraxanthin also demonstrated favorable binding energies, particularly against BACE-1 (−12.7 and −12.4 kcal/mol, respectively), while 3-hydroxy-β-apo-8′-carotenoic acid showed more balanced but slightly weaker affinities across the panel. Collectively, these findings support the hypothesis that specific carotenoids can act as broad-spectrum inhibitors of key AD–related proteins, potentially addressing the multifactorial pathology of the disease.

**Figure 2:**
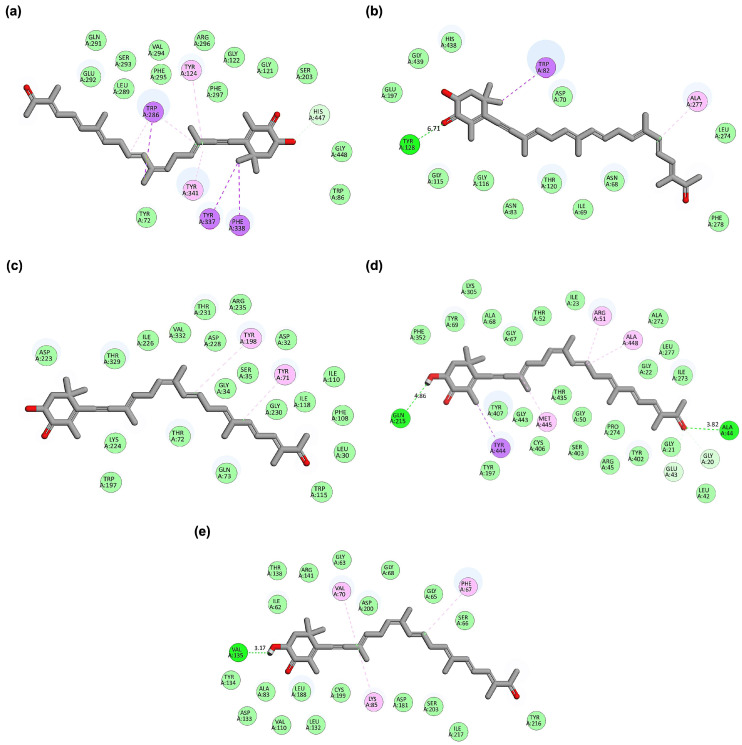
2D interaction maps of the best-performing carotenoid (Hopkinsiaxanthin). (**a**) Hopkinsiaxanthin_AChE complex. (**b**) Hopkinsiaxanthin_BChE complex. (**c**) Hopkinsiaxanthin_BACE-1 complex. (**d**) Hopkinsiaxanthin_MAO complex. (**e**) Hopkinsiaxanthin_GSK3-β complex. The interaction types are color-coded as follows: hydrogen bonds (bright green), carbon-hydrogen bonds (light green), van der Waals interactions (pale green), Pi-Alkyl (pink), and Pi-Sigma (purple). Red color indicates oxygen atoms in the functional groups.

Binding pose and interaction analysis further elucidated the molecular mechanisms underlying these docking results (Figure 2). Hopkinsiaxanthin interacted with AChE predominantly through hydrophobic contacts involving key aromatic residues (TYR337, PHE338, TRP286, TYR124, TYR341), complemented by a hydrogen bond with HIS447, stabilizing its position within the active gorge (Figure 2a). In the BChE complex, the compound engaged in hydrophobic interactions with TRP82 and ALA277 and formed a hydrogen bond with TYR128, reflecting binding consistency across cholinesterase targets (Figure 2b). Against BACE-1, the interactions were dominated by π-π stacking and hydrophobic contacts with TYR71 and TYR198, residues critical for substrate recognition, suggesting the carotenoid could effectively interfere with amyloidogenic processing (Figure 2c).

In the MAO complex, Hopkinsiaxanthin demonstrated extensive hydrophobic interactions with TYR444, PHE352, and TYR407, accompanied by three hydrogen bonds (ALA44, GLN215, GLY20) (Figure 2d). These interactions collectively contributed to its exceptionally low binding energy (−12.6 kcal/mol), highlighting MAO inhibition as a potential mechanism for neuroprotection. Finally, the GSK3-β complex revealed hydrophobic contacts with VAL70, LYS85, and PHE67, alongside a stabilizing hydrogen bond with VAL135 (Figure 2e). Interestingly, an unfavorable donor–acceptor interaction with VAL135 was also observed, which may slightly reduce binding stability but does not negate the overall strong affinity (−9.0 kcal/mol). These results highlight Hopkinsiaxanthin as a potent multi-target ligand, capable of simultaneously engaging multiple proteins central to AD pathology. Its dual mode of interactions, hydrophobic stacking with aromatic residues and stabilizing hydrogen bonds, provides structural versatility, enabling it to adapt across diverse binding pockets. The favorable docking energies and their ADMET-supported pharmacokinetics reinforce the therapeutic promise of carotenoids, particularly Hopkinsiaxanthin, as natural scaffolds for multi-target drug development in AD.

**Figure 3:**
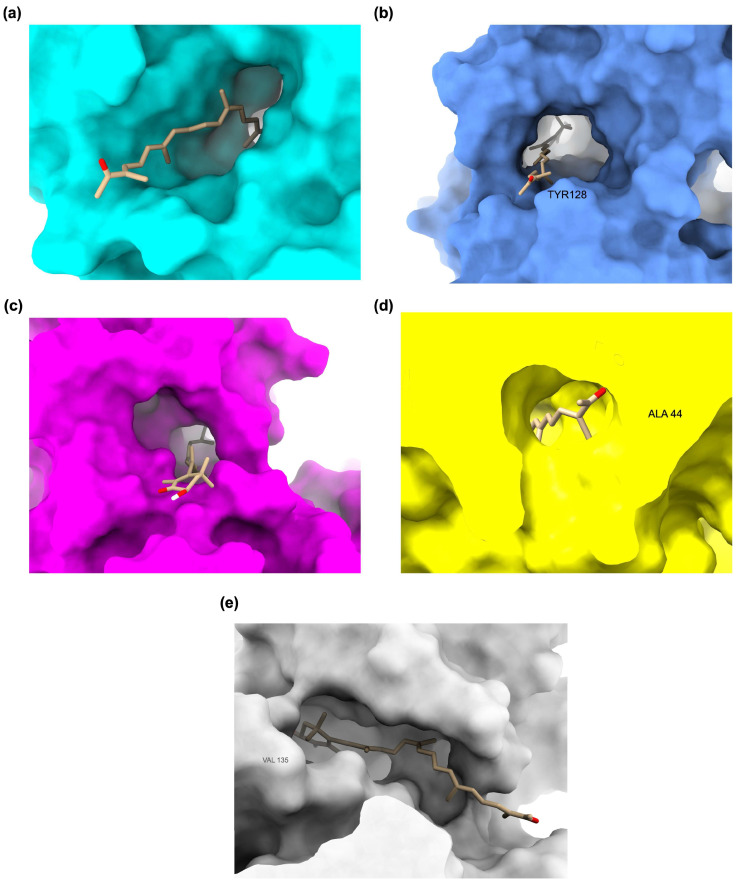
Comparative 3D binding poses of the best-performing carotenoid (Hopkinsiaxanthin) in the AD–related receptor’s ligand-binding domain (LBD). Protein surfaces are shown to illustrate cavity topology and global steric complementarity, while the ligand is displayed in stick representation. Labels indicate representative residues near the binding region for spatial reference. Detailed residue-level interaction analyses are provided in subsequent interaction maps and energy decomposition figures. (**a**) Hopkinsiaxanthin_AChE complex (cyan). (**b**) Hopkinsiaxanthin_BChE complex (cornflower blue). (**c**) Hopkinsiaxanthin_BACE-1 complex (magenta). (**d**) Hopkinsiaxanthin_MAO complex (yellow). (**e**) Hopkinsiaxanthin_GSK3-β complex (light gray). Red color indicates oxygen atoms in the functional groups.

The 3D docking visualizations highlight how Hopkinsiaxanthin adapts within the binding cavities of AD–related receptors. Figure 3 provides a comparative surface representation of the ligand embedded within the catalytic or ligand-binding domains (LBDs) of each receptor, emphasizing global shape complementarity rather than detailed residue-by-residue interactions. In the AChE complex (Figure 3a), Hopkinsiaxanthin aligned stably within the catalytic gorge, with its elongated conjugated backbone fitting the hydrophobic channel. The visualization shows deep insertion along the aromatic gorge that connects the peripheral anionic site to the catalytic active site, illustrating geometric complementarity with the tunnel-like cavity. This orientation rationalizes its strong affinity and resemblance to the binding pattern of standard AChE inhibitors. For BChE (Figure 3b), the compound occupied the enzyme’s cavity in a conformation that maximizes hydrogen bonding and hydrophobic stabilization. The broader active-site pocket of BChE is visibly accommodated by slight conformational adjustment of the polyene chain, demonstrating structural adaptability. This explains its favorable binding energy and potential as a dual cholinesterase inhibitor.

Within the BACE-1 active site (Figure 3c), Hopkinsiaxanthin positioned itself deep in the substrate-binding cleft, predominantly stabilized by hydrophobic forces. The surface representation highlights enclosure within the large catalytic pocket characteristic of aspartyl proteases, supporting steric compatibility with the binding groove. The snug fit across this large pocket suggests its ability to block amyloid-β formation effectively. In the MAO complex (Figure 3d), the ligand extended along the catalytic channel, engaging through a combination of hydrogen bonding and hydrophobic contacts. The elongated topology of the MAO cavity is visually apparent, with Hopkinsiaxanthin spanning the channel-like pocket in a manner consistent with known MAO ligands. This balance of interactions contributes to its stability within the site and supports its role in modulating oxidative stress pathways. For GSK3-β (Figure 3e), Hopkinsiaxanthin spanned across the ATP-binding pocket in a manner consistent with kinase inhibitors, maintaining stable contacts despite minor unfavorable interactions. The surface view illustrates occupation of the ATP cleft region, emphasizing steric complementarity within the kinase catalytic core. This orientation highlights its potential to interfere with tau hyperphosphorylation mechanisms. Overall, the 3D binding analyses confirm Hopkinsiaxanthin’s versatility in adapting to diverse protein environments. Its elongated, hydrophobic-rich structure allows flexible accommodation across multiple active sites, while maintaining strong stabilization.

### 3.4. Frontier Molecular Orbital (HOMO–LUMO) Results

**Figure 4:**
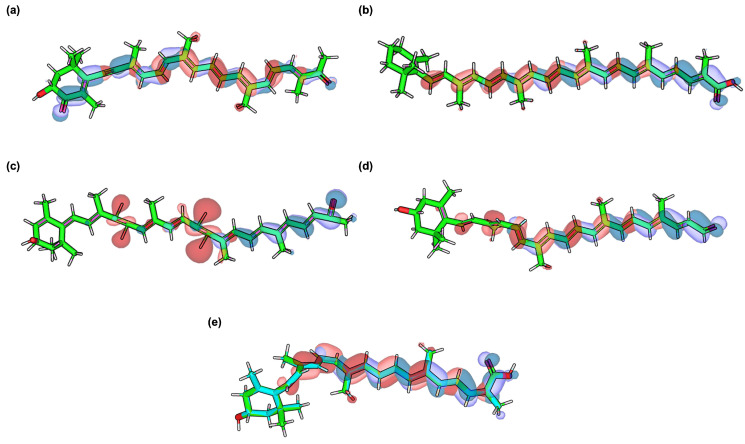
HOMO–LUMO profiles of top-performing carotenoids. (**a**) Hopkinsiaxanthin. (**b**) Neurosporaxanthin. (**c**) Tangeraxanthin. (**d**) Aesculaxanthin. (**e**) 3-Hydroxy-β-apo-8′-carotnoic acid. Blue regions represent HOMO localization, while red regions denote LUMO distribution.

The frontier molecular orbital (FMO) analysis was carried out to examine the electronic distribution and reactivity patterns of the top-performing carotenoids. Figure 4 illustrates the spatial localization of the highest occupied molecular orbital (HOMO) and lowest unoccupied molecular orbital (LUMO), while Table 3 provides their corresponding orbital energies, HOMO–LUMO gaps, and dipole moments. The results offer a deeper understanding of the carotenoids’ ability to participate in electron transfer processes, which are crucial in modulating redox-related pathways implicated in AD. Hopkinsiaxanthin exhibited a HOMO energy of −5.52 eV and a LUMO energy of −2.17 eV, resulting in a gap of 3.35 eV. The HOMO density was mainly localized along the polyene chain, suggesting a propensity for electron donation, while the LUMO distribution extended across the terminal groups, indicating favorable sites for electron acceptance. This balance of orbital localization and a moderate gap size supports its stability and reactivity, aligning well with its strong binding affinities observed in docking and MD simulations.

Neurosporaxanthin displayed a slightly lower gap (3.04 eV) due to its relatively higher HOMO energy (−5.07 eV), which enhances electron-donating ability. Its moderate dipole moment (3.57 D) suggests good electronic polarization, allowing adaptability in diverse protein microenvironments. Similarly, Aesculaxanthin showed a gap of 3.16 eV with a dipole moment of 6.14 D, reflecting both stability and potential for polar interactions, which may contribute to its strong complementarity with hydrophilic residues. Among the candidates, Tangeraxanthin presented the narrowest gap (2.02 eV), indicative of high electronic reactivity. The delocalization of the HOMO and LUMO across its conjugated backbone facilitates efficient charge transfer, making it potentially more reactive under oxidative stress conditions. However, its high dipole moment (9.57 D) may also confer increased polarity, which could influence solubility and membrane permeability in biological systems. In contrast, 3-hydroxy-β-apo-8′-carotenoic acid showed the largest HOMO–LUMO gap (3.43 eV), corresponding to higher stability but lower electronic reactivity. Its relatively low dipole moment (2.43 D) suggests weaker polar interactions, which might limit adaptability in binding pockets compared to other carotenoids. Nevertheless, the balance between stability and reduced reactivity may provide protective benefits against undesired oxidative degradation.

### 3.5. Pharmacophore Modeling Results

**Figure 5:**
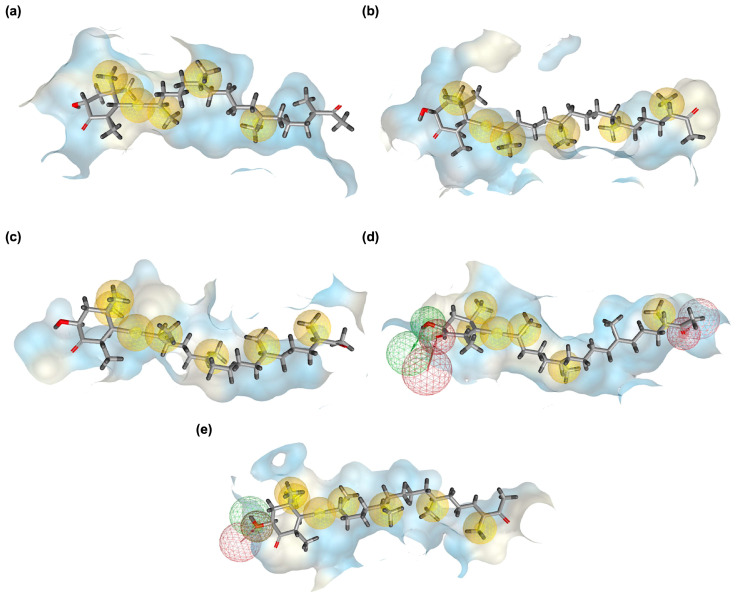
3D pharmacophore modeling. (**a**) Hopkinsiaxanthin_AChE complex. (**b**) Hopkinsiaxanthin_BChE complex. (**c**) Hopkinsiaxanthin_BACE-1 complex. (**d**) Hopkinsiaxanthin_MAO complex. (**e**) Hopkinsiaxanthin_GSK3-β complex. Yellow spheres indicate hydrophobic interaction centers; green directional features represent hydrogen bond donor projections; red directional features signify hydrogen bond acceptor projections; and semi-transparent mesh spheres (“excluded volumes”) denote sterically restricted regions of the binding pocket. These excluded volumes define spatial constraints that guide ligand optimization and prevent steric clashes.

Pharmacophore modeling was employed to further rationalize the docking and FMO results by identifying the essential molecular features governing recognition between Hopkinsiaxanthin and AD–related targets. As shown in Figure 5, the pharmacophore maps highlighted a combination of hydrophobic contacts, hydrogen bond donors, and hydrogen bond acceptors, which collectively underpin ligand binding and stability within the receptor environments. This integrative approach allows the visualization of conserved interaction motifs consistent with Hopkinsiaxanthin’s structural and electronic properties. For the Hopkinsiaxanthin–AChE complex (Figure 5a), the pharmacophore profile was dominated by extensive hydrophobic features along the polyene chain. These interactions support efficient stabilization within the lipophilic binding gorge of AChE, complementing the docking results that revealed strong van der Waals contacts. The absence of significant hydrogen bonding further emphasizes that π–alkyl and hydrophobic contacts are the principal driving forces of binding.

The Hopkinsiaxanthin–BChE complex (Figure 5b) also exhibited a similar pattern, with hydrophobic spheres distributed along the conjugated backbone. However, a subtle hydrogen bond acceptor feature suggests an additional stabilizing interaction, potentially contributing to its comparable affinity to AChE. These findings indicate that Hopkinsiaxanthin can adapt its interaction mode within structurally similar but distinct cholinesterase active sites. In the case of BACE-1 (Figure 5c), hydrophobic interactions again dominated, but they were more dispersed throughout the binding cavity. This distribution reflects the adaptability of Hopkinsiaxanthin’s extended polyene structure to occupy a large catalytic cleft. The pharmacophore model supports the docking evidence that carotenoids can act as non-competitive binders by engaging multiple hydrophobic hotspots rather than relying on specific hydrogen-bonding interactions. A different profile was observed in the MAO complex (Figure 5d), where hydrogen bond donor and acceptor features were mapped in addition to hydrophobic contacts. The combined presence of these features suggests that Hopkinsiaxanthin may form polar contacts with residues near the entrance of the active site while maintaining hydrophobic anchoring within the core pocket. This mixed pharmacophore pattern aligns with the dual necessity of hydrophobic stabilization and polar complementarity for effective MAO inhibition. Finally, in the GSK3-β complex (Figure 5e), hydrophobic features were again predominant, with two additional hydrogen bond donor and acceptor regions localized near the terminal groups of Hopkinsiaxanthin. This suggests that while hydrophobic contacts drive primary stabilization, polar interactions may fine-tune ligand orientation, contributing to the moderate affinity observed in docking. Such a pharmacophore profile highlights the potential of Hopkinsiaxanthin to interfere with kinase function via multi-point interactions.

In Figure 5, yellow spheres represent hydrophobic interaction centers mapped along the conjugated polyene backbone; green directional features correspond to hydrogen bond donor projections; red directional features denote hydrogen bond acceptor projections; and the semi-transparent mesh spheres (“net spheres”) represent excluded volume constraints that define steric boundaries of the binding pocket. These excluded volumes are critical because they indicate spatial regions inaccessible to ligand atoms, thereby guiding rational modification of the scaffold without steric clashes. This abstraction allows identification of conserved hydrophobic corridors within elongated binding clefts (e.g., AChE gorge and GSK3-β ATP pocket) that are less obvious when viewing only atomistic docking poses. Importantly, pharmacophore modeling was not intended to replicate the docking poses shown in Figure 2, but rather to abstract and spatially generalize the key interaction features responsible for ligand recognition across different targets. While Figure 2 presents atomistic binding orientations within specific protein environments, Figure 5 extracts recurring interaction motifs (hydrophobic regions, hydrogen bond donor/acceptor sites, and steric constraints) into a simplified 3D feature map that can be used for scaffold interpretation, comparative analysis, and future virtual screening efforts. Thus, the pharmacophore modeling lies in transforming structural poses into transferable interaction patterns rather than providing redundant structural visualization.

### 3.6. MD Simulations Reveal Structural Stability and Interaction Profiles

**Figure 6:**
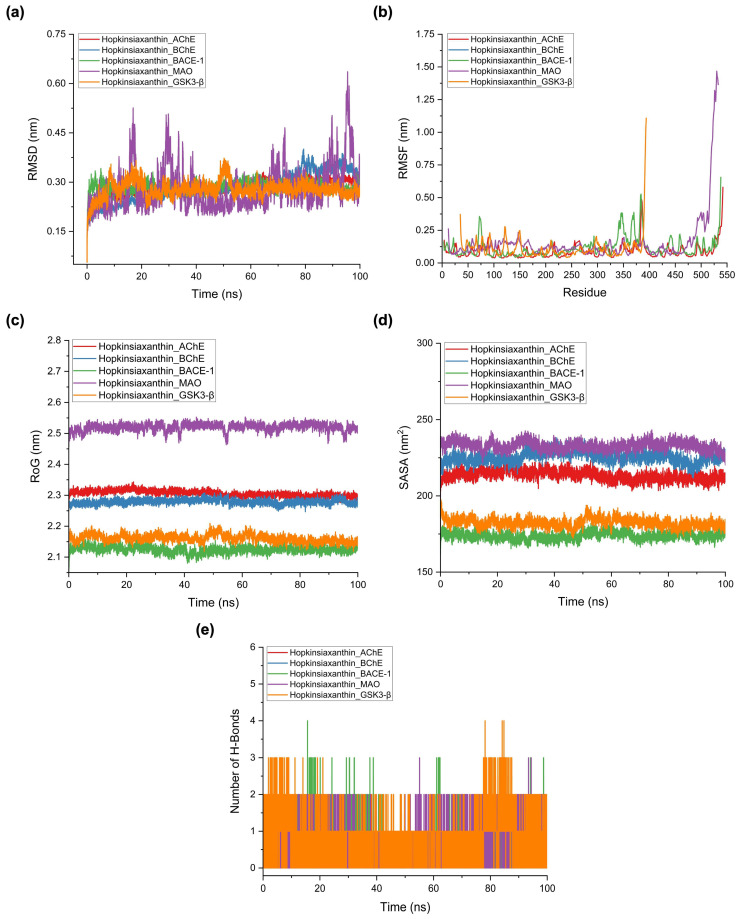
MD simulation results for AD–related receptors complex with Hopkinsiaxanthin over 100 ns of simulation. (**a**) Root mean square deviation (RMSD), reflecting the overall conformational stability of the protein–ligand complexes. (**b**) Root mean square fluctuation (RMSF) provides residue-level insights into backbone flexibility, particularly in the active-site regions. (**c**) Radius of gyration (RoG), indicating the degree of compactness and folding stability of the protein throughout the trajectory. (**d**) Solvent accessible surface area (SASA), showing changes in surface exposure and solvation upon ligand binding. (**e**) The number of hydrogen bonds represents the occupancy and stability of polar contacts sustaining protein–ligand interactions.

The MD simulations were performed to further validate the stability and dynamic behavior of the Hopkinsiaxanthin complexes with AD–related targets. The root mean square deviation (RMSD) trajectories (Figure 6a) demonstrated that all receptor–ligand systems attained equilibrium within the first 10–15 ns and remained stable over the 100 ns production run. The average RMSD values ranged between 0.278 and 0.285 nm (Table 4), underscoring the absence of significant structural drift. Among the systems, the Hopkinsiaxanthin_MAO complex displayed slightly higher RMSD fluctuations compared to the others, which can be attributed to the larger catalytic pocket of MAO that permits greater conformational plasticity and transient repositioning of the ligand. In contrast, the Hopkinsiaxanthin_GSK3-β complex exhibited the lowest deviation, indicating a snug ligand fit that restricted backbone fluctuations and promoted long-term conformational stability. This behavior is particularly significant because GSK3-β inhibition is considered one of the crucial therapeutic strategies for slowing tau hyperphosphorylation in AD. These results confirm that Hopkinsiaxanthin can be accommodated in structurally diverse receptor environments while preserving overall protein integrity, highlighting its potential as a multi-target agent. The root mean square fluctuation (RMSF) analysis (Figure 6b) offered detailed residue-level insights into protein flexibility and dynamic adaptations upon ligand binding. Across all complexes, the average atomic fluctuations were below 0.2 nm, suggesting minimal destabilization of the protein backbone. However, localized differences were observed near the ligand-binding clefts. The MAO complex exhibited the most pronounced residue fluctuations (0.158 ± 0.194 nm), reflecting enhanced flexibility of loop regions surrounding the active site. Such behavior suggests an induced-fit mechanism, in which the protein pocket adapts dynamically to optimize ligand accommodation and interaction stability. In contrast, AChE and BChE displayed relatively rigid binding-site dynamics, consistent with the structural constraints of their narrow hydrophobic gorges. This rigidity aligns with the docking results, where hydrophobic contacts dominated the interaction landscape. It indicates that once Hopkinsiaxanthin enters these pockets, it stabilizes within a confined environment that restricts conformational rearrangements. Interestingly, BACE-1 and GSK3-β exhibited intermediate flexibility profiles, balancing backbone rigidity with localized adaptability, which may facilitate strong binding affinity and stable long-term occupancy. These RMSF findings collectively reinforce the notion that Hopkinsiaxanthin leverages induced-fit flexibility (as in MAO) and rigid-site stabilization (as in AChE and BChE) to establish robust interactions with multiple AD targets.

The analysis of the radius of gyration (RoG) (Figure 6c) demonstrated that all protein–ligand complexes retained a compact structural state throughout the 100 ns simulation. Average RoG values ranged from 2.124 nm in the BACE-1 complex to 2.519 nm in the MAO complex, reflecting inherent differences in protein size, structural domains, and folding compactness (Table 4). Importantly, none of the trajectories showed sharp fluctuations or long-term drifts, suggesting that ligand binding did not induce partial unfolding or destabilization of the global protein architecture. The relatively small standard deviations associated with the RoG values further support this conclusion, indicating consistent packing stability of the receptor backbones. The largest RoG value observed in the MAO complex is consistent with its broader catalytic pocket and higher RMSFs, which collectively reflect a more flexible topology. Conversely, the tightest packing observed in the BACE-1 complex suggests that Hopkinsiaxanthin binding enhances the overall folding stability of this protease, potentially limiting unnecessary conformational rearrangements that could compromise substrate recognition.

The solvent-accessible surface area (SASA) analysis (Figure 6d) provided additional insights into solvation and conformational adaptability of the complexes. Among the systems, the MAO complex displayed the highest SASA value (232.885 ± 3.053 nm^2^), in line with its larger active site cavity and dynamic solvent exposure. This elevated SASA suggests that Hopkinsiaxanthin binding does not significantly shield the surface from solvent but promotes a partially solvent-accessible conformation that could facilitate transient interactions with surrounding water molecules or co-factors. On the other hand, the BACE-1 complex exhibited the lowest SASA (173.869 ± 2.491 nm^2^), consistent with its deep and relatively buried binding pocket. Reduced SASA values typically reflect enhanced hydrophobic burial and compact packing, which can contribute to stronger ligand retention and diminished solvent-mediated destabilization. AChE, BChE, and GSK3-β complexes demonstrated intermediate SASA values, maintaining relatively constant profiles throughout the trajectory. These observations highlight how intrinsic receptor topologies modulate the solvation environment of Hopkinsiaxanthin, which may influence ligand residence times and ultimately pharmacological efficacy.

Hydrogen bond analysis (Figure 6e) revealed that although polar contacts were modest in number, they played an essential role in stabilizing specific complexes. On average, hydrogen bond occupancies ranged from as low as 0.064 in the BChE complex to as high as 0.901 in the GSK3-β complex (Table 4). The consistently higher occupancy of hydrogen bonds in the GSK3-β and MAO systems suggests that polar interactions are particularly relevant in stabilizing these targets, complementing the hydrophobic and π–π stacking contacts identified during docking and pharmacophore analyses. Interestingly, the relatively low hydrogen bond counts in AChE and BChE suggest that these systems rely more heavily on nonpolar interactions within their narrow hydrophobic gorges, where water exclusion enhances van der Waals stabilization. Although hydrogen bonds were not the dominant interaction force across the systems, their contribution is crucial for strengthening binding specificity and orientation fidelity, thereby preventing ligand dissociation during long-term simulations. The combination of hydrophobic enclosure and intermittent hydrogen bonding thus supports a dual stabilization mechanism by which Hopkinsiaxanthin maintains strong yet adaptable binding across diverse AD targets.

### 3.7. MM/PBSA Free Energy Analysis and Per-Residue Decomposition

To further validate the stability and thermodynamic feasibility of Hopkinsiaxanthin binding to AD–related receptors, MM/PBSA free energy calculations were conducted over the equilibrated 100 ns trajectories. As shown in Table 5, all complexes displayed favorable binding free energies, ranging from −17.14 to −22.73 kcal/mol, indicating thermodynamically favorable ligand–receptor interactions within the sampled conformational ensemble. We emphasize that MM/PBSA estimates reflect relative binding free energies under equilibrium conditions and do not provide information about binding kinetics, transition-state barriers, or full entropic contributions to the association process. Among the systems, Hopkinsiaxanthin_GSK3-β exhibited the strongest binding affinity (−22.73 ± 5.19 kcal/mol), followed closely by Hopkinsiaxanthin_AChE (−21.50 ± 6.03 kcal/mol). In contrast, Hopkinsiaxanthin_BACE-1 presented the weakest binding energy (−17.14 ± 6.94 kcal/mol), although still indicative of a stable and energetically favorable interaction. These results are consistent with the docking scores, where GSK3-β and AChE were predicted as primary high-affinity targets. However, the present calculations do not allow conclusions regarding the kinetic accessibility of binding or the height of potential activation barriers separating bound and unbound states.

**Figure 7:**
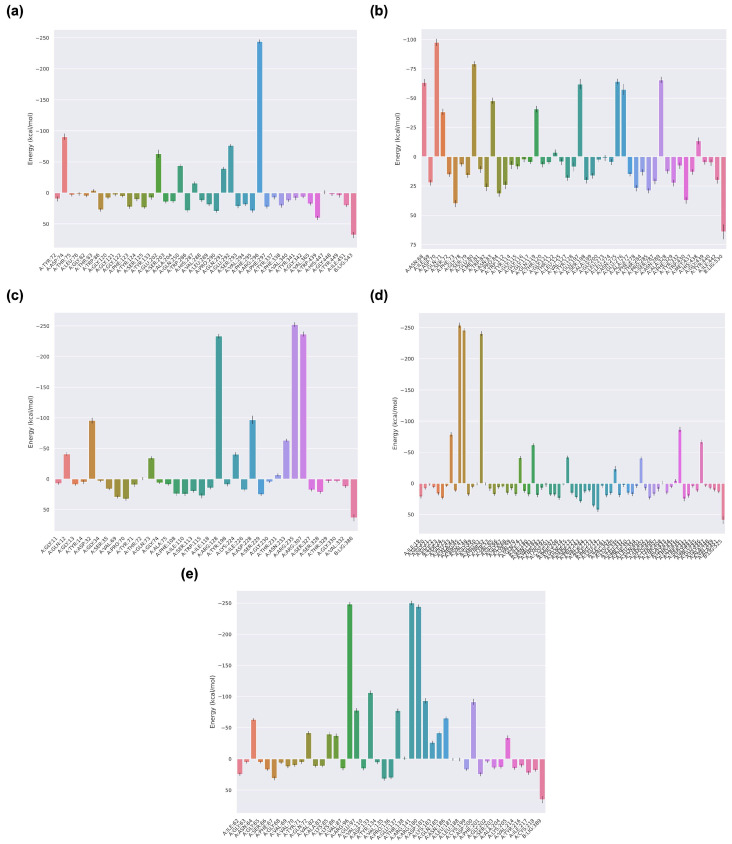
Per-residue energy contributions (kcal/mol) of Hopkinsiaxanthin in complex with AD–related receptors. (**a**) Hopkinsiaxanthin_AChE complex. (**b**) Hopkinsiaxanthin_BChE complex. (**c**) Hopkinsiaxanthin_BACE-1 complex. (**d**) Hopkinsiaxanthin_MAO complex. (**e**) Hopkinsiaxanthin_GSK3-β complex. Bars represent individual residue contributions to the binding free energy, where negative values indicate favorable (stabilizing) interactions and positive values indicate unfavorable contributions. The color gradient reflects residue sequence position (for visual separation of residue groups) rather than physicochemical classification.

The per-residue energy decomposition analysis (Figure 7a–e) provided molecular-level insights into the specific amino acids contributing to ligand stabilization. It should be noted that per-residue decomposition reflects energetic contributions within the MM/PBSA framework and does not explicitly separate enthalpic and entropic components. In the AChE complex (Figure 7a), residues ASP74, GLU202, GLN250, GLN291, GLU292, and ARG296 contributed strongly to favorable binding. These residues are located near the catalytic anionic site and peripheral anionic site, which were highlighted in docking as key anchoring regions. Their contributions reflect a mixture of electrostatic stabilization from acidic residues and hydrogen bonding from polar side chains, consistent with the docking-derived interactions of Hopkinsiaxanthin with the hydrophobic gorge. For the BChE complex (Figure 7b), major contributors included ASN86, ASP70, GLN71, GLU80, and SER198, along with residues in the mid-gorge region such as ASN275 and GLU276. These interactions align with docking predictions, where Hopkinsiaxanthin formed both polar and hydrophobic contacts along the catalytic gorge. Importantly, SER198 belongs to the catalytic triad of BChE, suggesting that ligand engagement near this site could contribute to inhibitory potential. The contributions of ALA328 and other gorge-lining residues also reflect the dominance of hydrophobic stabilization, consistent with the relatively low hydrogen bond occupancy observed in MD simulations.

In the BACE-1 complex (Figure 7c), residues ASP32, ASP228, ARG235, and ARG307 were identified as the strongest contributors, forming an electrostatic network characteristic of aspartyl protease active sites. Docking results had also highlighted hydrogen bonding and ionic interactions with the catalytic dyad ASP32/ASP228, which are critical for ligand anchoring. Despite these favorable hotspots, the overall ΔG_binding was weaker compared to other targets, likely due to higher flexibility observed in RMSF analyses, which may introduce entropic effects not fully captured in the present MM/PBSA treatment. The MAO complex (Figure 7d) demonstrated stabilization driven by residues GLU43, ARG47, VAL48, THR52, and TRP441. The involvement of hydrophobic residues (VAL48, ILE77, and VAL182) alongside polar contributors highlights the mixed nature of Hopkinsiaxanthin–MAO interactions. Notably, TRP441, previously identified in docking as a key π–π stacking partner, made a substantial energetic contribution in the MM/PBSA profile, reinforcing the role of aromatic contacts in ligand recognition. These results corroborate the higher solvent-accessible surface area observed in MD simulations, which may permit flexible ligand accommodation while maintaining strong localized interactions. Finally, the GSK3-β complex (Figure 7e) displayed the most extensive network of favorable energetic contributions, with key residues including ASN64, GLN72, ARG96, GLU97, ASP133, GLU137, ARG141, and LYS183. These residues cluster within the ATP-binding cleft, confirming docking predictions that Hopkinsiaxanthin targets the kinase’s catalytic core. The dominance of electrostatic contributors such as ASP133, GLU137, and ARG141 explains the strong ΔG_binding observed, further stabilized by hydrogen bonding identified during MD simulations. This convergence of docking and free energy decomposition supports GSK3-β as a particularly favorable molecular target for Hopkinsiaxanthin, with the potential to disrupt pathological phosphorylation cascades relevant to AD progression. Nevertheless, experimental kinetic assays and enhanced sampling simulations would be required to determine association/dissociation rates and fully resolve the thermodynamic landscape of binding.

## 4. Discussion

This study screened a large, structurally diverse library of carotenoids (n = 1191). It used a stepwise computational screening workflow (ADMET, FMO analysis, molecular docking, pharmacophore modeling, MD simulation, and MM/PBSA calculations) to identify and characterize multi-target inhibitors for AD. Our principal finding is that Hopkinsiaxanthin consistently ranks among the top compounds by docking affinity, MD stability, and MM/PBSA binding energy across five validated AD targets (AChE, BChE, BACE-1, MAO, and GSK3-β). The concordance between docking, dynamic simulations, and free-energy estimates gives confidence that Hopkinsiaxanthin’s interactions are both geometrically plausible and thermodynamically favorable, a pattern that has been reported for other natural carotenoids with neuroprotective potential (e.g., astaxanthin) and supports the general idea that carotenoids can serve as multitarget neuroprotective scaffolds [72]. AD is a multifactorial disease where cholinergic deficit, Aβ generation, monoaminergic imbalance, oxidative stress, and tau pathology all contribute to neurodegeneration. Therefore, a multi-target directed ligand that engages several of these nodes simultaneously is attractive and in line with recent therapeutic strategies advocating polypharmacology for complex CNS disorders. Our results show Hopkinsiaxanthin engaging cholinesterases (AChE/BChE), BACE-1, MAO, and GSK3-β with favorable docking scores and negative MM/PBSA ΔG_binding values, indicating potential to modulate neurotransmission, amyloidogenic processing, oxidative/deaminative pathways, and tau-kinase activity within a single scaffold. This multi-target concept is consistent with current literature that suggests natural polyenes and other natural products can act on multiple AD-relevant targets and pathways [73,74].

A strength of our pipeline is the agreement between static docking predictions and dynamic stability/energetics from MD and MM/PBSA. Hopkinsiaxanthin displayed low (more favorable) docking ΔG values for AChE and MAO, and these translated into stable MD trajectories (low RMSD, limited RoG drift) and substantial MM/PBSA binding free energies (e.g., −21.50 kcal·mol^−1^ for AChE, −18.78 kcal·mol^−1^ for MAO). Per-residue decomposition identified complementary hot-spots (acidic and aromatic residues in AChE; aromatic and hydrophobic residues in MAO and GSK3-β) that align with the 2D interaction maps and pharmacophore features (hydrophobic chains + occasional H-bond anchors). In Figure 7, the color gradient does not represent residue physicochemical classes; instead, it reflects the sequential position of residues along the protein (i.e., a visual grouping to distinguish residue indices across the binding pocket). This interpretation is supported by the consistent left-to-right color progression observed across all panels. Regarding the apparent predominance of positive energy contributions, it is important to clarify the sign convention used in MM/PBSA per-residue decomposition. Negative values correspond to favorable (stabilizing) contributions to ligand binding, whereas positive values represent unfavorable (destabilizing) contributions. Although several residues exhibit positive contributions, this is expected in protein–ligand systems due to local strain, desolvation penalties, or suboptimal contacts. Crucially, binding affinity is governed by the net sum of all residue contributions, and in our study, the total ΔG_binding values remain negative, confirming overall favorable binding. Thus, the coexistence of unfavorable per-residue contributions does not contradict the stability of the complexes but instead reflects a realistic energetic balance within the binding pocket. Moreover, key hotspot residues with large negative contributions dominate the energetic landscape, outweighing smaller unfavorable contributions and driving the overall binding process. This pattern is commonly observed in MM/PBSA analyses, where a limited number of residues contribute disproportionately to binding stabilization. Similar multi-method cross-validation has been used effectively in prior natural-product docking studies to prioritize leads and to rationalize mechanisms of action for carotenoids and polyphenols [75,76].

FMO (HOMO–LUMO) analysis of the top carotenoids provided additional mechanistic context. We acknowledge that the FMO calculations were performed on isolated ligands optimized in the gas phase and therefore represent a single conformational and electronic reference state. These calculations were not used as quantitative predictors of binding affinity nor integrated directly into the docking scoring function. Instead, they were employed comparatively to characterize global electronic properties across top-ranked carotenoids and to support post-docking prioritization. Hopkinsiaxanthin’s moderate HOMO–LUMO gap (3.35 eV) and HOMO localization along the polyene chain suggest an ability to donate electron density into aromatic or polar protein microenvironments while remaining chemically stable. We considered redox-related electronic properties relevant because oxidative stress is a central pathogenic mechanism in Alzheimer’s disease, contributing to cholinergic dysfunction, amyloid-β aggregation, and kinase dysregulation [77]. In particular, monoamine oxidase activity directly generates hydrogen peroxide during neurotransmitter deamination, linking enzyme function to local ROS production [78]. Carotenoids are well-established chain-breaking antioxidants capable of quenching singlet oxygen and scavenging peroxyl radicals due to their extended conjugated π-system [79,80]. Experimental studies further indicate that carotenoids such as astaxanthin can attenuate oxidative stress and modulate AD–related pathways, including cholinesterase activity and amyloid toxicity [81]. Thus, our hypothesis is that a balanced redox profile, reflected by a moderate HOMO–LUMO gap, may complement classical binding interactions by stabilizing oxidative microenvironments surrounding AD-relevant enzymes, rather than acting as a standalone inhibitory mechanism. Although Hopkinsiaxanthin adopted different conformations upon docking, its extended conjugated backbone remained electronically delocalized across poses, meaning that the qualitative distribution of frontier orbitals is expected to be largely preserved despite torsional adjustments. Thus, the FMO descriptors were interpreted as global electronic fingerprints of the scaffold rather than conformation-specific interaction energies. By contrast, compounds with minimal gaps (higher reactivity) could be prone to non-specific oxidation or instability. In contrast, large gaps indicate low reactivity and potentially weaker electronic complementarity with catalytic residues. In this way, FMO analysis served as a qualitative electronic stability/reactivity filter and mechanistic interpretative tool, rather than a standalone quantitative binding metric. Prior studies have emphasized the importance of conjugated electronic structure in carotenoid antioxidant and protein interaction behavior; our FMO data are concordant with that literature [32].

A consistent ADMET challenge for carotenoids is their high lipophilicity (LogP often > 5), which our screening also revealed. High LogP can favor membrane partitioning and intestinal permeability but compromises aqueous solubility and can complicate oral formulation and systemic delivery. Our representative set (including Hopkinsiaxanthin) showed strong predicted intestinal absorption and high Caco-2 permeability (a good sign for oral uptake), but modest BBB metrics (logBB values around −0.1 to −0.4). Literature on other carotenoids suggests that some (e.g., astaxanthin, fucoxanthin) cross the BBB in vivo and exert central effects, but crossing efficiency is variable and often dose-/formulation-dependent. Thus, the translational route for Hopkinsiaxanthin would likely require optimized formulations (nanoemulsions, liposomal or lipid-based carriers, prodrugs) to ensure adequate brain exposure while preserving favorable safety. These formulation strategies have precedent in the carotenoid literature and are consistent with recommendations from pharmacokinetics studies of hydrophobic nutraceuticals [72,82]. Encouragingly, predicted toxicity metrics for the representative carotenoids were favorable (high LD50, negative AMES prediction, no hepatotoxicity or skin sensitization), and none were predicted to be CYP3A4 or CYP2D6 inhibitors (although many are CYP3A4 substrates). This metabolic profile suggests moderate clearance without a high risk of CYP-mediated drug interactions, a common issue with lipophilic natural products. Nonetheless, in vitro metabolic stability assays and human microsome studies will be required because in silico CYP predictions are not definitive; conversion to metabolites could alter activity or safety. Similar studies on other carotenoids underscore the importance of profiling Phase I/II metabolites early in development [83]. Several experimental studies report neuroprotective, anti-amyloid, and anti-inflammatory effects for carotenoids such as astaxanthin and fucoxanthin in cellular and animal AD models; some of these compounds have demonstrated BBB penetration and cognitive benefits in vivo. Our computational results are consistent with that body of evidence and extend it by showing that specific carotenoids (Hopkinsiaxanthin in our case) may act simultaneously on several enzymatic targets implicated in AD pathology. This attribute could potentiate disease-modifying rather than purely symptomatic effects. However, it is essential to emphasize that in silico potency does not guarantee in vivo efficacy: bioavailability, metabolism, off-target effects, and blood–brain delivery remain critical obstacles that require experimental validation [72,84].

## 5. Limitations and Future Works

Although this study provides an in-depth computational evaluation of Hopkinsiaxanthin as a multi-target therapeutic candidate for AD, several limitations should be acknowledged. First, molecular docking and MM/PBSA free energy estimations, while powerful, are still approximations that do not fully account for entropic contributions, long-timescale conformational changes, or solvent effects beyond the simulated window. Similarly, our 100 ns molecular dynamics simulations capture intermediate conformational stability but may miss slower allosteric transitions or ligand-induced folding events that could influence binding affinity and selectivity. Second, the ADMET predictions relied entirely on in silico models, which, although validated on large datasets, may not accurately reflect true pharmacokinetics, bioavailability, or toxicity in vivo. In particular, the high lipophilicity (LogP > 5) observed in Hopkinsiaxanthin may pose challenges for solubility and blood–brain barrier penetration, which could reduce its translational viability despite favorable computational predictions. Finally, while per-residue decomposition provided valuable insights into potential hotspot interactions, it does not substitute for experimental binding or crystallographic validation to confirm the structural determinants of ligand recognition.

Future studies should therefore pursue a multi-pronged validation approach. Biochemically, Hopkinsiaxanthin should be tested in vitro against the major AD–related targets identified here (AChE, BChE, BACE-1, MAO, and GSK3-β) to determine its real binding affinities and inhibitory kinetics. Structural methods such as X-ray crystallography or cryo-EM could further validate the docking-derived binding modes and confirm interaction networks. From a pharmacological perspective, cell-based assays are required to evaluate its neuroprotective efficacy in models of oxidative stress, Aβ aggregation, and tau hyperphosphorylation, alongside cytotoxicity and BBB permeability assays to assess safety and CNS accessibility. In vivo validation using animal models of AD will be critical to determine pharmacokinetics, brain bioavailability, and therapeutic outcomes. Moreover, formulation optimization (e.g., lipid-based carriers, nanoemulsions, or prodrug strategies) could improve solubility and enhance brain delivery, addressing one of the main translational barriers of highly lipophilic carotenoids. Taken together, these steps will be essential to bridge the gap between promising computational predictions and the clinical potential of Hopkinsiaxanthin as a multi-target directed agent for AD.

## 6. Conclusions

In conclusion, this study employed an integrative in silico framework that combined ADMET predictions, molecular docking, FMO analysis, pharmacophore modeling, MD simulations, and MM/PBSA free energy calculations to systematically evaluate Hopkinsiaxanthin as a computationally prioritized multitarget candidate against AD. Across AChE, BChE, BACE-1, MAO, and GSK3-β, Hopkinsiaxanthin demonstrated stable binding orientations, favorable estimated binding affinities and interaction energy profiles, and complementary pharmacophoric features that collectively suggest a potential capacity to modulate diverse AD–related pathways. The MD simulation outcomes, including consistent RMSD, RoG, SASA, and hydrogen bond stability, highlighted the structural integrity of receptor–ligand complexes, suggesting that Hopkinsiaxanthin does not destabilize the protein backbone while maintaining specific polar and hydrophobic interactions. Furthermore, MM/PBSA decomposition revealed critical residues contributing significantly to binding, aligning well with molecular docking predictions and reinforcing the robustness of the computational approach. While ADMET analyses pointed to potential challenges in solubility and blood–brain barrier penetration, the overall results position Hopkinsiaxanthin as a promising in silico lead scaffold, warranting further investigation, rather than a confirmed therapeutic agent. Importantly, all findings presented here are based on computational modeling and should be interpreted as hypothesis-generating. Experimental validation through in vitro enzymatic assays, cellular studies, pharmacokinetic profiling, and in vivo models will be essential to confirm biological activity, safety, and therapeutic relevance. These findings provide mechanistic insights into ligand–target interactions and establish a preliminary computational foundation for subsequent experimental and formulation-based investigations aimed at validating and optimizing Hopkinsiaxanthin for potential therapeutic development.

## Figures and Tables

**Table 1:** Representative of carotenoid compounds analyzed in this study, with their SMILES notations and 2D structural depictions. The complete set of 1191 carotenoid compounds is provided in Supplementary Data S1. Red color indicates oxygen atoms in the functional groups.

Carotenoid	SMILES	2D Structure
Hopkinsiaxanthin	C1C(C(=O)C(=C(C1(C)C)C#C/C(=C/C=C/C(=C/C=C/C=C(/C=C/C=C(\C)/C(=O)C)\C)/C)/C)C)O	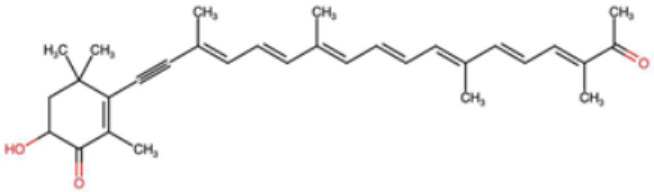
Neurosporaxanthin	C1CCC(=C(C1(C)C)/C=C/C(=C/C=C/C(=C/C=C/C=C(/C=C/C=C(/C=C/C=C(/C(=O)O)\C)\C)\C)/C)/C)C	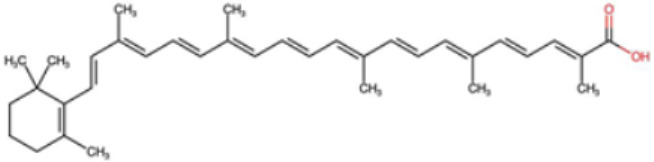
Tangeraxanthin	C1=C(/C(=C/C=C(/C=C/C=C(/C=C/C=C/C(=C/C=C/C(=C/C=C/C(=O)C)/C)/C)\C)\C)/C(CC1O)(C)C)C	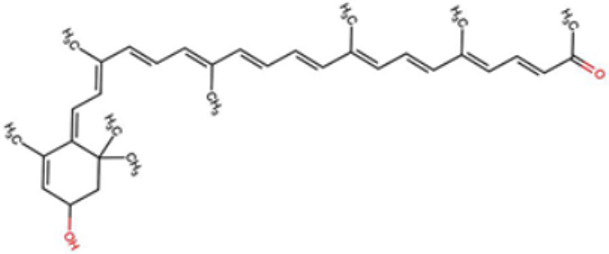
Aesculaxanthin	C1[C@@H](CC(=C(C1(C)C)/C=C/C(=C/C=C/C(=C/C=C/C=C(/C=C/C=C(/C=C/C=O)\C)\C)/C)/C)C)O	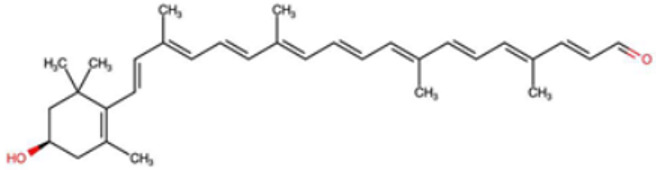
3-Hydroxy-β-apo-8′-carotnoic acid	OC(=O)C(C)=C/C=C/C(C)=C/C=C/C=C(\C)C=C/C=C(\C)C=C/C1=C(\C)C[C@](O)([H])CC1(C)C	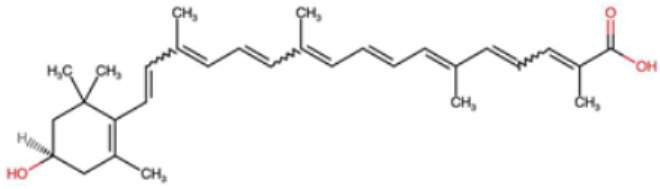
3-Hydroxysintaxanthin	C1[C@@H](CC(=C(C1(C)C)/C=C/C(=C/C=C/C(=C/C=C/C=C(/C=C/C=C(/C(=O)C)\C)\C)/C)/C)C)O	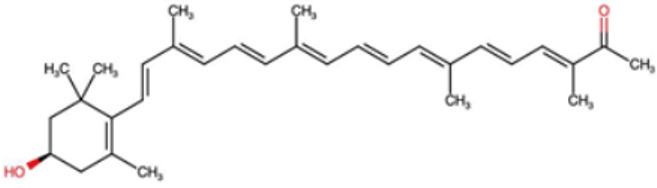
Apoastacenal	C1=C(C(=O)C(=C(C1(C)C)/C=C/C(=C/C=C/C(=C/C=C/C=C(/C=C/C=C(\C)/C=O)\C)/C)/C)C)O	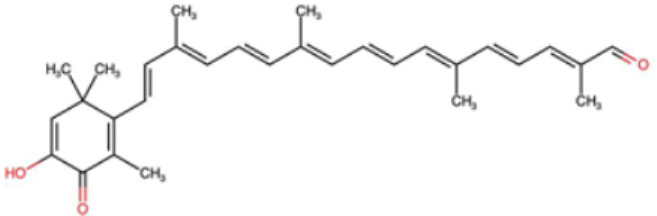
Cochloxanthin	C1C(=O)C=C(C(C1(C)C)(/C=C/C(=C/C=C/C(=C/C=C/C=C(/C=C/C=C(/C(=O)O)\C)\C)/C)/C)O)C	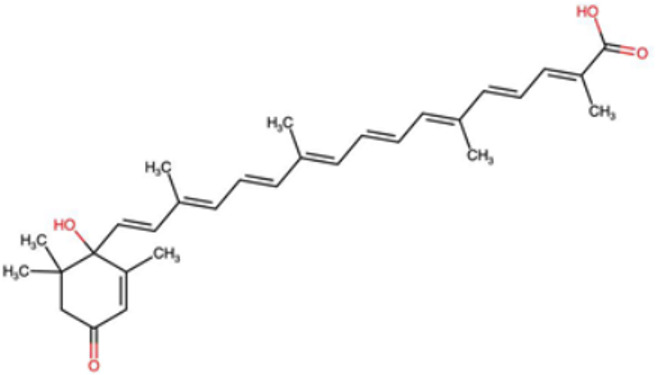
Triophaxanthin	CC(=O)/C(=C/C=C/C(=C/C=C/C=C(/C=C/C=C(/C#CC1=C(C[C@H](CC1(C)C)O)C)\C)\C)/C)/C	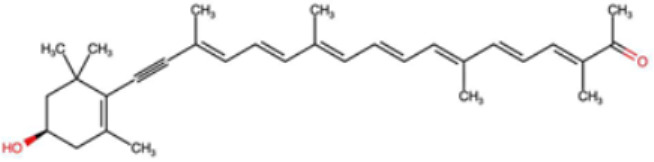
12′-Apoastaxanthinal	C1[C@@H](C(=O)C(=C(C1(C)C)/C=C/C(=C/C=C/C(=C/C=C/C=C(/C=O)\C)/C)/C)C)O	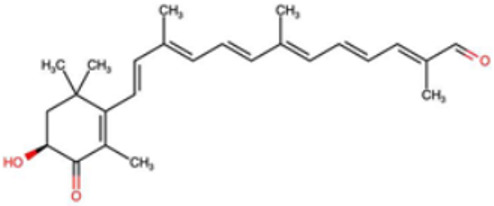

**Table 2:** In silico ADMET parameters of top-performing carotenoids suitable for central nervous system (CNS) drug candidacy.

Parameter	Hopkinsiaxanthin	Neurosporaxanthin	Tangeraxanthin	Aesculaxanthin	3-Hydroxy-β-apo-8′-carotnoic Acid
Molecular weight (MW)	458.642	498.751	484.724	458.686	448.647
LogP	6.709	10.110	8.805	8.248	7.578
Hydrogen bond acceptors (HBA)	3	1	2	2	2
Hydrogen bond acceptors (HBD)	1	1	1	1	2
Caco2 permeability	1.43	1.177	1.197	1.198	1.372
Intestinal absorption	94.41	92.167	91.216	91.857	91.356
BBB permeability	−0.114	−0.069	−0.214	−0.199	−0.382
CNS permeability	−1.542	−1.277	−1.354	−1.409	−1.610
CYP2D6 substrate	No	No	No	No	No
CYP3A4 substrate	Yes	Yes	Yes	Yes	Yes
CYP2D6 inhibitior	No	No	No	No	No
CYP3A4 inhibitior	No	No	No	No	No
Total clearance	1.163	1.308	1.365	1.411	1.282
Median lethal dose (LD_50_)	2.073	1.816	2.241	1.762	2.478
AMES toxicity	No	No	No	No	No
Hepatotoxicity	No	No	No	No	No
Skin sensitisation	No	No	No	No	No

**Table 3:** Frontier molecular orbital energies, HOMO–LUMO gap, and dipole moments of the top-performing carotenoids.

Molecule	HOMO (eV)	LUMO (eV)	Gap (eV)	Dipole (D)
Hopkinsiaxanthin	−5.52	−2.17	3.35	5.48
Neurosporaxanthin	−5.07	−2.03	3.04	3.57
Tangeraxanthin	−4.96	−2.94	2.02	9.57
Aesculaxanthin	−5.30	−2.14	3.16	6.14
3-Hydroxy-β-apo-8′carotnoic acid	−5.18	−1.76	3.43	2.43

**Table 4:** MD simulation parameters of AD–related receptors complex with Hopkinsiaxanthin over 100 ns of simulations, including RMSD, RMSF, RoG, SASA, and number of hydrogen bond interactions.

Complex	Average RMSD (nm)	Average RMSF (nm)	Average RoG (nm)	Average SASA (nm^2^)	Number of Hydrogen Bonds Between the Ligand-Receptor
Hopkinsiaxanthin_AChE	0.285 ± 0.023	0.087 ± 0.057	2.306 ± 0.009	213.451 ± 3.186	0.161 ± 0.372
Hopkinsiaxanthin_BChE	0.283 ± 0.044	0.117 ± 0.077	2.278 ± 0.008	225.615 ± 3.711	0.064 ± 0.248
Hopkinsiaxanthin_BACE-1	0.284 ± 0.015	0.117 ± 0.062	2.124 ± 0.011	173.869 ± 2.491	0.485 ± 0.639
Hopkinsiaxanthin_MAO	0.279 ± 0.066	0.158 ± 0.194	2.519 ± 0.012	232.885 ± 3.053	0.577 ± 0.660
Hopkinsiaxanthin_GSK3-β	0.278 ± 0.025	0.112 ± 0.099	2.161 ± 0.012	182.409 ± 2.898	0.901 ± 0.665

**Table 5:** MM/PBSA binding free energies (ΔG_binding) of Hopkinsiaxanthin in complex with AD–related receptors.

Complex	MM/PBSA Free Binding Energy ΔG_binding (kcal/mol)
Hopkinsiaxanthin_AChE	−21.50 ± 6.03
Hopkinsiaxanthin_BChE	−19.74 ± 3.55
Hopkinsiaxanthin_BACE-1	−17.14 ± 6.94
Hopkinsiaxanthin_MAO	−18.78 ± 6.79
Hopkinsiaxanthin_GSK3-β	−22.73 ± 5.19

## Data Availability

The original contributions presented in this study are included in the article/Supplementary Materials. Further inquiries can be directed to the corresponding authors.

## Supplementary Materials

The following supporting information can be downloaded at: https://www.mdpi.com/article/10.3390/cimb48040407/s1.

## Abbreviations

The following abbreviations are used in this manuscript:

AChE	Acetylcholinesterase
AD	Alzheimer’s disease
ADMET	Absorption, distribution, metabolism, excretion, and toxicity
AIRs	Ambiguous interaction restraints
APP	Amyloid precursor protein
BACE-1	β-site amyloid precursor protein cleaving enzyme 1
BBB	Blood–brain barrier
BChE	Butyrylcholinesterase
CNS	Central nervous system
CYP	Cytochrome P450
DFT	Density functional theory
FMO	Frontier molecular orbital
GAFF2	General amber force field
GSK3-β	Glycogen synthase kinase-3 beta
HADDOCK	High ambiguity driven protein-protein docking
HBA	Hydrogen bond acceptor
HBD	Hydrogen bond donor
HIA	Human intestinal absorption
HOMO	Highest occupied molecular orbital
LBD	Ligand-binding domain
LogP	Lipophilicity
LUMO	Lowest unoccupied molecular orbital
MAO	Monoamine oxidase
MCI	Mild cognitive impairment
MD	Molecular dynamics
MM/PBSA	Molecular mechanics/Poisson-Boltzmann surface area
NPT	Number of particles, pressure, and temperature
NVT	Number of particles, volume, and temperature
PME	Particle mesh Ewald
PRODIGY	Protein binding energy prediction
RMSD	Root mean square deviation
RMSF	Root mean square fluctuation
RoG	Radius of gyration
ROS	Reactive oxygen species
SASA	Solvent-accessible surface area
SCF	Self-consistent field
SPCE	Single point charge extended
SPR	Surface plasmon resonance
STP	Single-trajectory protocol
TPSA	Topological polar surface area
